# Behcet's disease presenting with cerebral vasculitis: a case report

**DOI:** 10.1186/1757-1626-2-7528

**Published:** 2009-05-26

**Authors:** Banu Turgut Ozturk, Refik Oltulu, Hurkan Kerimoglu, Suleyman Okudan

**Affiliations:** Department of Ophthalmology, Meram Faculty of Medicine, Selcuk UniversityTurkey

## Abstract

**Introduction:**

Behcet's disease encompasses a group of multisystemic complications secondary to occlusive vasculitis. It presents usually with oral or genital ulcers however, other system involvements may be the first sign as well.

**Case presentation:**

A 26-years-old man admitted to our clinic for his decreased visual acuity. Ophthalmologic examination revealed bilateral vitritis and optic disc swelling. However, the meticulously taken history included other complains like headache and oral ulcers. Further investigation with cranial MRI demonstrated cerebral vasculitis secondary to Behcet's disease.

**Conclusion:**

Behcet's disease may be easy to diagnose when it presents with oral and genital ulcers or panuveitis, however presentation with optic disc swelling may warrant a thorough investigation.

## Introduction

Behçet's Disease (BD) is a chronic, multisystemic, inflammatory disorder characterized by intraocular inflammation, oral and mucosal ulcerations, skin lesions, and a variety of other disorders [1]. This disease with distinctive prevalence along the Silk Route and between the ages of 20-35 years is of utmost importance for the ophthalmologists as 50-80% of cases presented ocular involvement. The chronic, recurrent uveitis affecting the anterior and posterior uveal tract usually bilateral is the major cause of morbidity. The posterior segment involvement is described as retinal vasculitis and accompanied generally by vitritis and macular edema [2].

Though optic disc edema is a frequent finding in BD, differential diagnosis between papillitis and papilledema is warranted and cases without uveitis should be suspected inevitably for central nervous system (CNS) involvement [3]. A young male diagnosed to have BD with both ocular and central nervous system involvements as the initial finding is presented herein.

## Case presentation

**Figure 1.**
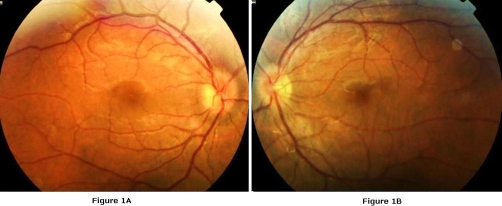
Fundus photograph of the case showing optic disc swelling. **(A)** Right eye, **(B)** Left eye.

**Figure 2.**
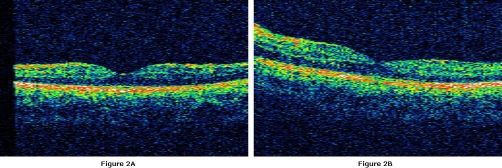
Macular images taken with optical coherens tomography. **(A)** Right eye. **(B)** Left eye.

**Figure 3.**
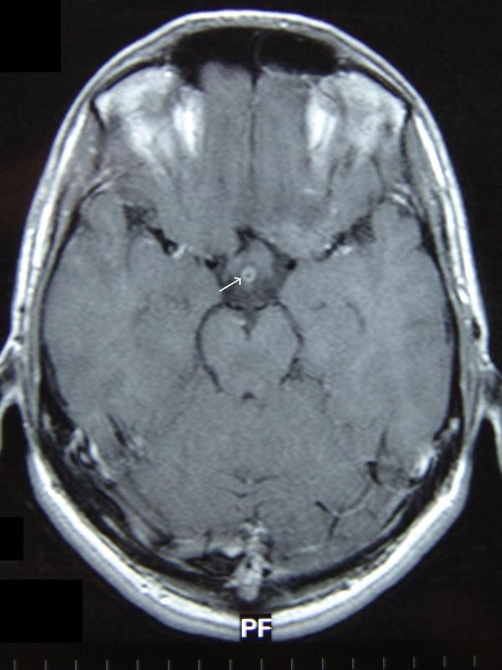
Magnetic resonance image demonstrating cerebral vasculitis.

A 26-years-old man was referred with decreased visual acuity for one week in the left eye. Headache was a accompanying complaint and he was overwhelmed by oral ulcers. On ophthalmologic examination, he had a visual acuity of 16/20 on both eyes. Color vision tested with Ishihara plates was 12/12. The anterior segment was normal and intraocular pressure measured with applanation tonometer revealed 12 mmHg on the right eye and 11 mmHg on the left eye. Fundus examination disclosed a mild vitritis (+1-2 cells) and optic disc swelling with absence of venous pulsation and cupping on both eyes (Figure 1A, 1B). As there was no sign of retinal infiltrates, vascular sheathing, engorgement, neovascularization, hemorrhages or cystoid macular edema (Figure 2A, 2B), a fluorescein angiography has not been performed. The central 30-2 test performed with Humphrey automatized perimetry demonstrated enlarged blind spot on the left eye and no abnormality on the right eye. Regarding the optic disc swelling and the complain of headache, a cranial magnetic resonance imaging (MRI) is planned which elicited a peripheral, ring-enhancing lesion with a diameter of 0.5 mm posterior to the optic chiasma on T1-weighted axial images obtained after administration of contrast agent (Figure 3). The lesion was interpreted as cerebral vasculitis by the neuro-radiologist. As the patient fulfilled the 3 major criteria required for the diagnosis [4] according to the examination in the dermatology clinic which revealed oral ulcer and acneiform lesions in addition to positive pathergy test; he was diagnosed to have Behcet's disease and oral treatment of methylprednisolone 1 mg/kg/day together with immunosuppressive therapy is offered. Optic disc swelling was assumed to be due to papilledema related to intracranial hypertension though it could not be proven as the patient denied lumbar puncture. As he had just a mild posterior uveitis we advised no further immunosuppressive drugs and just close follow-up, however Colchium-Dispert was suggested by the dermatology department. Unfortunately, the patient was not able to attend for further controls, so he was referred to another clinic with a detailed epicrisis.

## Discussion

BD is a chronic, relapsing, occlusive vasculitis of unknown etiology, affecting almost every organ system in the body. Though involvement of CNS is less common compared to ocular involvement, it may lead to lethal complications. Its prevalence is reported to be 2.2-4.9%, rising up to 5-10% in larger series and to 20% in autopsy series [3,5,6]. On the other hand, the incidence of initial involvement at the time of diagnosis is 5% for CNS and 20% for the eye according to various studies [7]. Ocular findings are frequently seen within the first years after diagnosis, while neurological findings are noticed 4-6 years after diagnosis, but the male predominance is noticed in both organ involvements [2,6].

Optic disc swelling is one of the frequent ocular findings in Behcet's disease and connotes papillitis in the first order, however bilateral cases with good visual acuity and lack of uveitis may be related to intracranial hypertension [8]. Therefore ocular findings presented in Table 1 like visual acuity, absence of venous pulsation, loss of cupping of the optic disc, pupillary reflexes, color vision, visual evoked potential should be evaluated precisely for the differential diagnosis between papillitis and papilledema (Table 1) [9].

**Table 1.** Differential Diagnosis of Optic Disc Swelling

	PAPILEDEMA	PAPILLITIS
Pain	Headache	Pain with eye movements
Laterality	Bilateral	Unilateral
Visual Acuity	Usually good	Diminished
Color Vision	Normal	Disturbed
Relative afferent pupil defect	Absent	Present
Vitreus cells	Absent	May be in posterior vitreus
Venous pulsation	Absent	Present
Optic disc cupping	Disappeared	Normal
Hemorrhages and exudates around the optic disc	Usually present	Rarely
Visual Field	Enlarged blind spot	Ceco-central scotoma

CNS involvement may be established in two different patterns in Behcet's disease: parenchymal and non-parenchymal. Parenchymal involvement is the most common form (82%) with a tendency to produce focal lesions clustering especially in the brainstem while the non-parenchymal type mainly result from vascular involvement and presents as dural sinus thrombosis, aseptic meningitis and arterial vasculitis. This type of manifestation is also called as vasculo-Behcet's disease and has a better prognosis [1,3]. Cerebral vasculitis is a rare type of non-parenchymal CNS involvement and MRI is reported to be highly sensitive and faithful imaging modality for diagnosis [10].

## Conclusion

Behcet's disease is characterized by occlusive vasculitis however presentation with vitritis and papilledema as the initial findings is not frequent [8]. On the other hand, differential diagnosis of cerebral vasculitis did not include Behcet's disease as the most frequent cause. Therefore taking the history of patients meticulously, especially in countries on the Silk Road, which are supposed to have a higher incidence of Behcet's disease, will prevent to overlook the supportive findings like uveitis, oral and genital ulcerations and enable to enlighten the dilemma rapidly and correctly.
